# Cucurbitacin B Exerts Antiaging Effects in Yeast by Regulating Autophagy and Oxidative Stress

**DOI:** 10.1155/2019/4517091

**Published:** 2019-06-02

**Authors:** Yanfei Lin, Yuki Kotakeyama, Jing Li, Yanjun Pan, Akira Matsuura, Yoshikazu Ohya, Minoru Yoshida, Lan Xiang, Jianhua Qi

**Affiliations:** ^1^College of Pharmaceutical Sciences, Zhejiang University, 866 Yu Hang Tang Road, Hangzhou, China; ^2^Departments of Integrated Biosciences, Graduate School of Frontier Sciences, University of Tokyo, 5-1-5 Kashiwanoha, Kashiwa, Chiba 277-8562, Japan; ^3^Department of Biology, Graduate School of Science, Chiba University, Chiba 263-8522, Japan; ^4^Chemical Genomics Research Group, RIKEN Center for Sustainable Resource Science, 2-1 Hirosawa, Wako, Saitama 351-0198, Japan; ^5^Department of Biotechnology and Collaborative Research Institute for Innovative Microbiology, The University of Tokyo, Yayoi 1-1-1, Bunkyo-ku, Tokyo 113-8657, Japan

## Abstract

The budding yeast *Saccharomyces cerevisiae* has been used as a model organism for the basic mechanism of aging, which provides useful assay systems for measuring both replicative and chronological lifespans. In the course of our screening program for substances that extend replicative lifespan, cucurbitacin B (CuB) was found as a hit compound from a compound library, which contains cerebrosides, phenols, sesquiterpenoid, triterpenoids, and sterols isolated from natural products by our research group. Importantly, it prolonged not only the replicative lifespan but also the chronological lifespan in yeast. CuB increased *ATG32* gene expression, suggesting that CuB induces autophagy. Indeed, the GFP signal generated from the cleavage of GFP-Atg8, which is a signature of autophagy, was increased upon CuB treatment. On the other hand, CuB failed to increase the chronological lifespans when either *ATG2* or *ATG32*, essential autophagy genes, was deleted, indicating that the lifespan extension by CuB depends on autophagy induction. Furthermore, CuB significantly increased superoxide dismutase (Sod) activity and the survival rate of yeast under oxidative stress, while it decreased the amount of reactive oxygen species (ROS) and malondialdehyde (MDA) production, indicating that CuB has activity to antagonize oxidative stress. Additionally, CuB did not affect replicative lifespans of *sod1*, *sod2*, *uth1*, and *skn7* mutants with the K6001 background, indicating that aging-related genes including *SOD1*, *SOD2*, *UTH1*, and *SKN7* participate in the antiaging effect of CuB. These results suggest that CuB exerts antiaging activity by regulating autophagy, ROS, antioxidative ability, and aging-related genes. Finally, we discuss the possible intracellular targets of CuB based on the phenotypic comparison between the CuB and global gene deletion databases.

## 1. Introduction

Yeast has the replicative lifespan and chronological lifespan. The replicative lifespan measures the number of daughters of a single mother cell, which can asexually produce prior to senescence. The chronological lifespan is defined as the length of time a yeast cell can survive in the nondividing G0 state [1]. Both of them are regulated by many environmental and genetic factors [2]. In our study, we used K6001 yeast as an aging model, which is a mutant yeast strain derived from W303. A unique character of K6001, in which only mother cells can produce daughter cells in glucose medium, but not in galactose medium [3], can be used for measuring replicative lifespan. Under the guidance of K6001 replicative lifespan assay, we have isolated several compounds with antiaging activity from natural products [4, 5].

Autophagy is a highly conserved pathway in organisms from yeast to human, which involves degradation of damaged organelles and proteins and circulation of amino acids and other metabolites [6]. It regulates the genomic integrity via suppression of cell division in yeast under starvation [7]. Besides, decreased autophagy leads to downregulation of proteostasis, which is one of the hallmarks for aging. Dysfunction or decreased expression of autophagy genes leads to shorter lifespan in yeast and fruit fly. Conversely, enhanced autophagy promotes the longevity in aging models and protects against aging and age-related disorders [8]. Specially, the lifespan-extending effects of rapamycin in *C. elegans*, fruit fly, and mice are abolished by knocking out or knocking down of *ATG* genes [9]. In addition, caloric restriction and resveratrol (RES) induced autophagy dependent on Sir2 function in yeast and *C. elegans*, and the deleted and silenced *ATG* genes avoid the antiaging effect of caloric restriction, RES, and overexpression of *SIR2* [10]. Therefore, autophagy has a close link to aging.

Oxidative stress is another cause of aging. Free radicals produced through aerobic metabolism accumulate over time and accelerate aging [11]. In yeast, the accumulation of ROS promotes replicative and chronological aging. The upregulation of antioxidant removes excess ROS and maintains redox balance, eventually delaying the progression of aging in yeast [12]. In our previous studies, we showed that antioxidative activity plays an important role in the antiaging effects of cholesterol and parishin [4, 5].

CuB has been shown as a promising compound for anticancer treatment in recent years, and it also shows antioxidative and anti-inflammatory activities [13, 14]. The regulation of STAT3 and Raf/MEK/ERK signaling pathways by CuB is likely to be involved in its antitumor activity such as induction of apoptosis in tumor cells [15]. Moreover, CuB was found to induce autophagy in cells [16]. In the present study, we identified CuB as a compound with antiaging activity in yeast, resulting from our screening for antiaging compounds from a compound library using yeast replicative and chronological lifespan assays. Here, we report a unique activity of CuB, which exerts antiaging effects in yeast through regulating autophagy and oxidative stress.

## 2. Materials and Methods

### 2.1. CuB, Yeast Strains, and Medium

**Figure 1:**
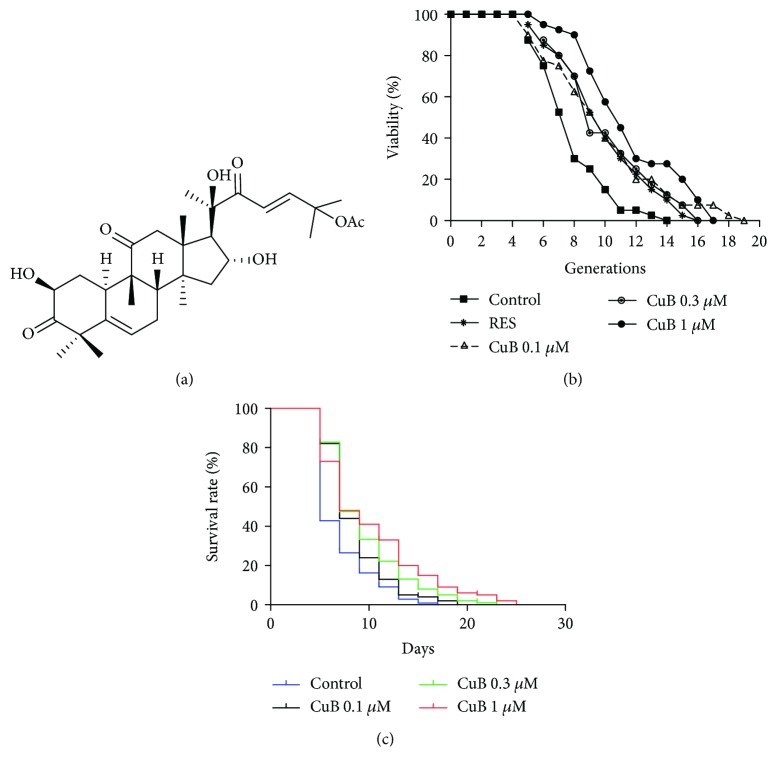
Chemical structure and antiaging activity of cucurbitacin B (CuB). (a) Chemical structure of CuB. (b) Effect of CuB on the replicative lifespan of K6001 yeast. RES (10 *μ*M) was used as the positive control. (c) Effect of CuB on the chronological lifespan of YOM36 yeast.

CuB was isolated from *Pedicellus melo* in accordance with a methodology that was previously used by our group. The chemical structure of CuB (Figure 1(a)) was identified by comparing HR ESI-MS, ^1^H NMR, and ^13^C NMR data with reported data [17]. ^1^H NMR (500 MHz, acetone-*d*_6_): *δ* 0.91 (3H, s), 1.02 (3H, s), 1.11 (1H, q, *J* = 13.0 Hz), 1.28 (3H, s), 1.32 (3H, s), 1.39 (3H, s), 1.40 (1H, m), 1.44 (3H, s), 1.51 (3H, s), 1.55 (3H, s), 1.84 (1H, m), 1.96 (3H, s), 1.97 (2H, m), 2.11 (1H, m), 2.40 (1H, dd, *J* = 5.5, 19.0 Hz), 2.51 (1H, d, *J* = 14.5 Hz), 2.66 (1H, d, *J* = 6.5 Hz), 3.02 (1H, d, *J* = 12.5 Hz), 3.40 (1H, d, *J* = 14.5 Hz), 4.45 (1H, m), 4.51 (1H, s), 4.56 (1H, m), 5.82 (1H, d, *J* = 3.0 Hz), 6.81 (1H, d, *J* = 16.0 Hz), and 6.98 (1H, d, *J* = 16.0 Hz); ^13^C NMR (125 MHz, acetone-*d*_6_): *δ* 19.2, 20.2, 20.6, 21.8, 22.0, 24.7, 25.1, 26.4, 27.1, 29.9, 34.2, 37.1, 43.6, 46.6, 49.0, 49.1, 49.4, 51.1, 51.4, 59.1, 71.4, 72.3, 79.6, 80.1, 120.8, 122.3, 142.0, 151.0, 170.3, 203.5, 212.7, and 213.7; and high-resolution ESI-TOF-MS *m/z* 581.3085, calcd for C_32_H_46_NaO_8_ (M+Na)^+^ 581.3090. All the yeast strains used in the present study are listed in Table 1.

### 2.2. Replicative and Chronological Lifespan Assays

The replicative lifespan assay was performed following a previously described methodology [5]. In brief, the K6001 strain was inoculated in galactose medium and cultured for 24–28 h in a shaking incubator at 160 rpm and 28°C. After washing with phosphate-buffered saline (PBS) for three times, approximately 4000 cells were spread onto glucose agar plates (2% glucose, 2% hipolypeptone, 1% yeast extract, and 2% agar) supplemented with 0, 0.1, 0.3, or 1 *μ*M CuB or 10 *μ*Μ RES. Afterward, the yeast cells were cultured at 28°C for 2 days. Forty microcolonies from each group were observed under microscopy, and the number of daughter cells in each microcolony was counted. The replicative lifespan assays of K6001 mutants (Δ*uth1*, Δ*skn7*, Δ*sod1*, Δ*sod2*, Δ*atg2*, and Δ*atg32* of K6001) were conducted similarly to those of the wild-type K6001. RES was procured from J&K Scientific Ltd. (Beijing, China) and was used as positive control.

The chronological lifespan assay was conducted in accordance with a previous methodology [18]. Briefly, YOM36 yeast cells were cultured in synthetic defined (SD) medium (0.17% yeast nitrogen base without amino acids and ammonium sulfate (BD Difco), 0.5% ammonium sulfate, and 0.2% glucose) for 24 h and then inoculated into the SD medium containing 0, 0.1, 0.3, or 1 *μ*M CuB with the initial OD600 value of 0.01. Cultures were grown in a shaker at 180 rpm and 30°C. Growth kinetics was recorded by measuring the OD600 value every 2 or 4 h until the stationary phase was reached. In addition, the survival rate was measured by counting colony-forming units (CFUs) every 2 days. The CFUs on day 3 was denoted as 100% survival.

### 2.3. Real-Time Polymerase Chain Reaction Analysis

Wild-type BY4741 were incubated with the negative control or 1 *μ*M CuB in glucose medium overnight. RNA was extracted through a hot phenol method. cDNA was synthesized through the reverse transcription method using the HiFi-MMLV cDNA Kit (CoWin Biotech, Beijing, China) and 5 *μ*g of RNA. Real-time polymerase chain reaction (RT-PCR) was performed in reference to a previous study [5] by using CFX96 Touch (Bio-Rad, Hercules, USA) and SYBR Premix Ex Taq (Takara, Otsu, Japan). The thermal cycling parameters were as follows: for *ATG2* and *ATG32*, 40 cycles, 94°C for 15 s, 51.6°C for 15 s, and 68°C for 20 s. The primers used for RT-PCR are as follows: for *ATG2*, sense 5′-GCT CCT GTC AGA TCG TTT AT-3′ and antisense 5′-TTC AGA CTC CTT CCC AAA TG-3′; for *ATG32*, sense 5′-ACC GTC TCA TCC CTT TAA AC-3′ and antisense 5′-CTT CCT CAA AAG CCT CAT CT-3′; and for *TUB1*, sense 5′-CCA AGG GCT ATT TAC GTG GA-3′ and antisense 5′-GGT GTA ATG GCC TCT TGC AT-3′. The 2^−ΔΔCt^ method was used to analyze relative gene expression data. The mRNA levels of *ATG2* and *ATG32* were normalized to those of *TUB1*.

### 2.4. GFP–ATG8 of Yeast Analyzed with Fluorescent Microscopy

YOM38 yeast cells containing pR316-GFP-ATG8 or YOM36 yeast cells were cultured in the SD medium with the initial OD600 value of 0.1 and treated with 0, 0.1, 0.3, or 1 *μ*M CuB or 300 *μ*M RES for 22 h. Subsequently, yeast cells were stained with 20 *μ*g/mL DAPI in dark for 10 min and washed with the PBS for three times. Yeast cells were observed using a two-photon confocal fluorescence microscope (Olympus FV1000BX-51, Tokyo, Japan) or fluorescent microscope (Leica DMI3000 B, Wetzlar, Germany). Pictures were acquired using image acquisition and analysis software.

### 2.5. Western Blot Analysis

YOM38 yeast cells containing the pR316-GFP-ATG8 plasmid were cultured as described in the previous section. At first, yeast cells were incubated with 300 *μ*M RES or CuB at doses of 0, 0.1, 0.3, or 1 *μ*M, and the yeast cells were collected at the specified time point and washed three times with PBS. After that, the samples were ultrasonicated for five times (1 min for each time), freeze-thawed for five times, and sonicated for five times again. The cell lysates were centrifuged, and the protein concentrations of the supernatant were measured with BCA Protein Assay Kit (CoWin Biotech, Beijing, China). Approximately 20 *μ*g protein was separated with SDS-PAGE and transferred to PVDF membranes. The membranes were incubated with primary antibodies followed by secondary antibodies. Antigens were visualized using ECL Western Blot Kit (CoWin Biotech, Beijing, China). The primary antibodies used are as follows: anti-GFP antibody (Medical & Biological Laboratories, Nagoya, Japan) and anti-*β*-actin antibody (CoWin Biotech, Beijing, China). The secondary antibodies used are as follows: horseradish peroxidase-linked anti-rabbit and anti-mouse IgGs (CoWin Biotech, Beijing, China).

### 2.6. Antioxidative Assay

BY4741 yeast cells with the initial OD600 value of 0.1 were cultured in liquid glucose medium and treated with 0, 0.1, 0.3, or 1 *μ*M CuB or 10 *μ*M RES for 24 h. Afterward, 5 *μ*L of yeast culture in each group with the same OD600 value was dropped onto glucose agar plates containing 9 mM H_2_O_2_. After 3 days, the growth of yeast was observed and photographed. Besides, further spots of 1/10, 1/100, and 1/1000 dilutions in each group were also dropped onto glucose agar plates containing 9 mM H_2_O_2_, and the growth was observed after 5 days.

Another method was performed to quantify the effect of CuB on the oxidative stress response of yeast. Similar to that in the former method, BY4741 yeast cells were treated with 0, 0.1, 0.3, or 1 *μ*M CuB or 10 *μ*M RES. 200 yeast cells from each group were spread onto glucose agar plates supplemented with or without 5 mM H_2_O_2_. The growth of yeast was observed after 2 days, and the number of microcolonies in each plate was counted. The survival rate was calculated as the ratio of the number of microcolonies in the presence of 5 mM H_2_O_2_ divided by the number of microcolonies in the absence of 5 mM H_2_O_2_.

### 2.7. ROS, MDA, and Sod Enzyme Assays

CuB- or RES-treated BY4741 yeast cells were cultured for 23 h in liquid glucose medium at 28°C. DCFH-DA (2′,7′-dichlorodihydrofluorescein diacetate, 1 *μ*L, 10 mM) was added to 1 mL of the cells. The mixture was then incubated at 28°C in the dark with shaking for 1 h. The cells were quickly washed with PBS thrice in the dark, and the DCF (2′,7′-dichlorofluorescein) fluorescence intensity of 1 × 10^7^ cells was recorded using the SpectraMax M3 multimode microplate reader (Molecular Devices Corporation, California, USA) under the excitation wavelength of 488 nm and emission wavelength of 525 nm.

BY4741 yeast cells were treated with CuB or RES in liquid glucose medium for 24 or 48 h at 28°C with the initial OD of 0.1. Yeast cells were collected through centrifugation and five cycles of ultrasonication (1 min for each time). Then, the cells were frozen for 5 min in liquid nitrogen, subsequently thawed for 2 min in a 37°C water bath, and sonicated for five cycles. The cell lysates were centrifuged, and the supernatant was removed for MDA quantification. MDA levels were quantified by using the MDA assay kit (Nanjing Jiancheng Bioengineering Institute, Nanjing, China) following the manufacturer's instructions.

BY4741 yeast cells were treated in a manner similar to that in MDA assay. Briefly, cells were treated with CuB or RES in the liquid glucose medium for 24 or 48 h at 28°C. Then, yeast cells were collected and subjected to five 1 min cycles of ultrasonication. Subsequently, the total super oxide dismutase (T-Sod) and Sod1 enzyme activities of the supernatant were tested using SOD Assay Kits (A001-1, A001-2) (Nanjing Jiancheng Bioengineering Institute, Nanjing, China) following the manufacturer's instructions.

### 2.8. Growth Curves of Yeast Cells with CuB

To test growth inhibition in liquid culture, the YPD medium containing 0, 1.56, 6.25, 25, or 50 *μ*M CuB was used. Cultures of Y02458, *his3∆* of BY4741 (*MATa*, *his3Δ1*, *leu2Δ0*, *met15Δ0*, *ura3Δ0*, and *YOR202w::KanMX4*) were treated with CuB and grown at 25°C for 25 h. Optical density from 0 to 25 hours was measured at 10 min intervals using an absorption spectrometer at 660 nm. The assay was repeated at least 2 times.

### 2.9. High-Dimensional Phenotypic Analysis

High-dimensional phenotypic analysis was conducted in accordance with a previous report [19]. Yeast cells were cultured in YPD medium with or without CuB, and cells in the logarithmic phase were fixed with 3.7% formaldehyde. Yeast cells, nuclear DNA, and actin were stained with 20 mg/mL fluorescein isothiocyanate-Con A, 4,6-diamidino-2-phenylindole, and rhodamine phalloidin, respectively. Morphological changes were observed through fluorescence microscopy. CalMorph was used to characterize each yeast cell through the quantification of 501 morphological parameters.

### 2.10. Fitness

To estimate fitness, we employed a previously published dataset of logarithmic strain growth rate coefficients for haploid nonessential gene deletion mutants grown on a basal medium (LSC basal) [20]. We used the *p* value calculated as the significance of lower fitness from the wild type of each strain based on one tail of the estimated probability distribution [20, 21] and calculated FDR as described previously [21].

### 2.11. Data Processing

Coefficient of variation values were highly dependent on the mean values in a nonlinear manner [22] and therefore were not suited for normalization. Instead, we defined noise values as the residuals between observed and predicted values, as described previously [21, 23].

### 2.12. Detection of Specific Morphologically Abnormal Mutants

The probability distribution of the wild-type replicates for each trait of the 501 parameters was estimated using maximum likelihood estimation with one of four probability density functions (gamma, beta, Gaussian, and beta-binomial distribution), as described previously [24]. We calculated the *p* value of every nonessential deletion mutant as morphological abnormality from the wild type for each trait (two-sided one-sample test) and identified the lowest *p* value among 501 traits as the “specific morphological abnormality,” as described previously [21]. Maximum likelihood estimation and calculation of the *p* value were performed using the gamlss function in R software's (http://www.r-project.org) gamlss package [25]. The FDR, a rate of type I errors in the rejected null hypothesis due to multiple comparisons, was calculated using the qvalue R function in the qvalue package [26]. We plotted specific morphologically abnormal mutants as circles filled with green.

### 2.13. Plot of Single-Gene Deletion Strains with Increased Lifespan

To plot single-gene deletion strains with increased lifespan, we employed a previously published dataset of replicative lifespan for nonessential gene deletion mutants [27].

### 2.14. Statistical Analysis

All statistical analyses were performed using GraphPad Prism 5 (GraphPad Software Inc.). Survival analysis was used for chronological lifespan assay. Analysis of variance was used to determine the significant differences among groups in all experiments, followed by two-tailed multiple *t*-tests with Bonferroni correction. A *p* value of less than 0.05 was considered statistically significant.

## 3. Results

### 3.1. CuB Has Significant Antiaging Effects

We have previously isolated a number of antiaging substances, such as cholesterol and parishin, from natural products on the basis of the K6001 lifespan assay [4, 5]. In the present study, we again used the K6001 lifespan assay to screen for antiaging compounds from a compound library and identified the active compound CuB (Figure 1(a)) as a potential antiaging compound. Subsequently, we performed chronological lifespan assay to confirm antiaging effects of CuB. As shown in Figure 1(b) and Table 2, the average replicative lifespan of each treatment group was as follows: 6.95 ± 0.34 generations for the control group, 9.03 ± 0.47 generations for the group treated with 10 *μ*M RES, 8.90 ± 0.55 generations for the group treated with 0.1 *μ*M CuB, 8.95 ± 0.51 generations for the group treated with 0.3 *μ*M CuB, and 9.60 ± 0.49 generations for the group treated with 1 *μ*M CuB. These results indicate that treatment with 0.1, 0.3, and 1 *μ*M CuB significantly prolongs the replicative lifespan of K6001 (*p* < 0.05, *p* < 0.05, and *p* < 0.01, respectively). As shown in Figure 1(c), CuB increased the survival rate of yeast (*p* < 0.001, *p* < 0.001, and *p* < 0.001, respectively). CuB significantly increased the days at which viability is equal to 0 of yeast from 15 ± 1.15 days to 17 ± 1.15, 21 ± 1.15, and 23.67 ± 0.67 days, respectively. These results suggest that CuB has significant antiaging effects on yeast cells.

### 3.2. CuB Regulates Autophagy in Yeast

**Figure 2:**
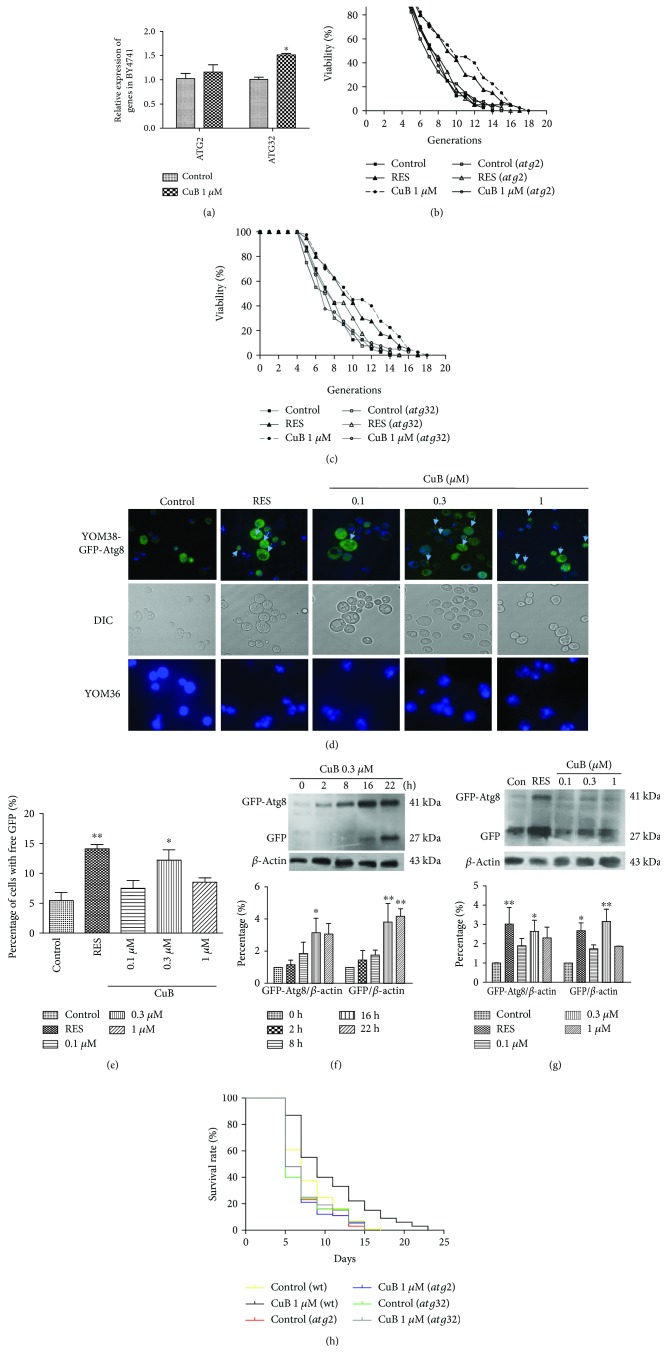
Effect of CuB on autophagy in yeast. (a) Effect of CuB on *ATG2* and *ATG32* gene expression after 12 h of treatment. (b, c) Effect of CuB on the replicative lifespan of K6001 and *atg2* (b) and *atg32* (c) mutants with K6001 background. (d) Fluorescent images of YOM38 yeast contained plasmid pR316-GFP-ATG8 and YOM36 yeast after treatment of RES or different doses of CuB in the SD medium and DAPI staining observed with a two-photon confocal fluorescent microscope. The upper line and middle line showed merged and DIC images of YOM38 yeast cells containing plasmid pR316-GFP-ATG8, respectively. The line below showed fluorescent images of YOM36. (e) Effect of CuB on the percentage of YOM38 cells containing plasmid pR316-GFP-ATG8 with free GFP. Three pictures containing more than 60 cells in each group were used for statistical analysis. (f) Western blot analysis of GFP-Atg8 and free GFP in yeast after shifting to the SD medium containing 0.3 *μ*M CuB for different times. (g) Western blot analysis of GFP-Atg8 and free GFP in yeast after treatment with RES or CuB for 22 h in the SD medium. (h) Effect of CuB on the chronological lifespan of wild-type YOM36 and *atg2* and *atg32* mutants with YOM36 background. ∗ and ∗∗ indicate significant difference between treatment groups and the corresponding control group (*p* < 0.05, *p* < 0.01).

Autophagy is a degenerative process that degrades cellular components for recycling into amino acids and other metabolites. It has essential roles in cell growth, differentiation, development, and aging [8]. Hence, we investigated the effect of CuB on autophagy. *ATG2* and *ATG32* are two of the most important genes that mediate autophagy in yeast; we first explored *ATG2* and *ATG32* gene expression in CuB-treated yeast. As shown in Figure 2(a), CuB significantly enhanced *ATG32* gene expression but did not significantly affect *ATG2* gene expression.

Furthermore, we constructed *atg2* and *atg32* mutants with K6001 background to do replicative lifespan assay. The changes of the lifespan are shown in Figures 2(b) and 2(c) and Table 2. The significant decrease in replicative lifespan of *atg2* and *atg32* mutants was not observed compared with that of K6001. After the treatment of CuB, the replicative lifespan of *atg2* and *atg32* mutants was also not affected. These results suggested that Atg2 and Atg32 did not take an important role in the replicative lifespan of yeast. However, they were required in the replicative lifespan extension of CuB.

To test whether CuB induces autophagy, we used the YOM38 strain, which expresses GFP-Atg8 at a physiological level and monitored the level of GFP upon treatment with CuB under fluorescent microscopy. The fluorescent images are displayed in Figure 2(d), and the statistic result is shown in Figure 2(e). CuB significantly enhanced the percentage of cells with green fluorescence at a dose of 0.3 *μ*M as does RES. In order to investigate whether the GFP signal came from autophagy or autofluorescence of yeast, the YOM36 yeast strain was also treated with RES or CuB. As shown in Figure 2(d), YOM36 expressed no green fluorescence. Besides, western blot analysis showed generation of free GFP, which is released into the vacuole during the autophagy flux. CuB increased the amount of released GFP in yeast in a time course manner, and 0.3 *μ*M CuB significantly increased the amount of free GFP (Figures 2(f) and 2(g)). These results confirm that CuB induces autophagy.

Furthermore, we performed chronological lifespan measurements using *atg2* and *atg32* mutants derived from a phototrophic derivative of BY4741 (YOM36) (Figure 2(h)). The results showed that the survival rate of *atg2* and *atg32* mutants significantly decreased (*p* < 0.05, *p* < 0.01) compared to YOM36 yeast strain. In addition, CuB at 1 *μ*M increased the survival rate of YOM36 yeast during the whole assay (*p* < 0.001), but failed to prolong the chronological lifespans of *atg2* and *atg32* mutants. The days at which viability is equal to 0 of *atg2* and *atg32* mutants were 14 ± 1 and 16 ± 1 days, respectively, and were shorter than those of the wild-type yeast (17 ± 0 days). In addition, CuB extended the days at which viability is equal to 0 of the wild-type yeast from 17 ± 0 days to 19 ± 2 days. However, the days at which viability is equal to 0 of the *atg2* and *atg32* mutants in CuB-treated groups were 13 ± 2 and 16 ± 1 days, respectively, indicating that CuB failed to prolong the chronological lifespans of *atg2* and *atg32* mutants. These results indicate that CuB exerts its activity through regulating autophagy, which requires *ATG2* and *ATG32*.

### 3.3. CuB Improves the Survival Rate of Yeast under Oxidative Conditions

**Figure 3:**
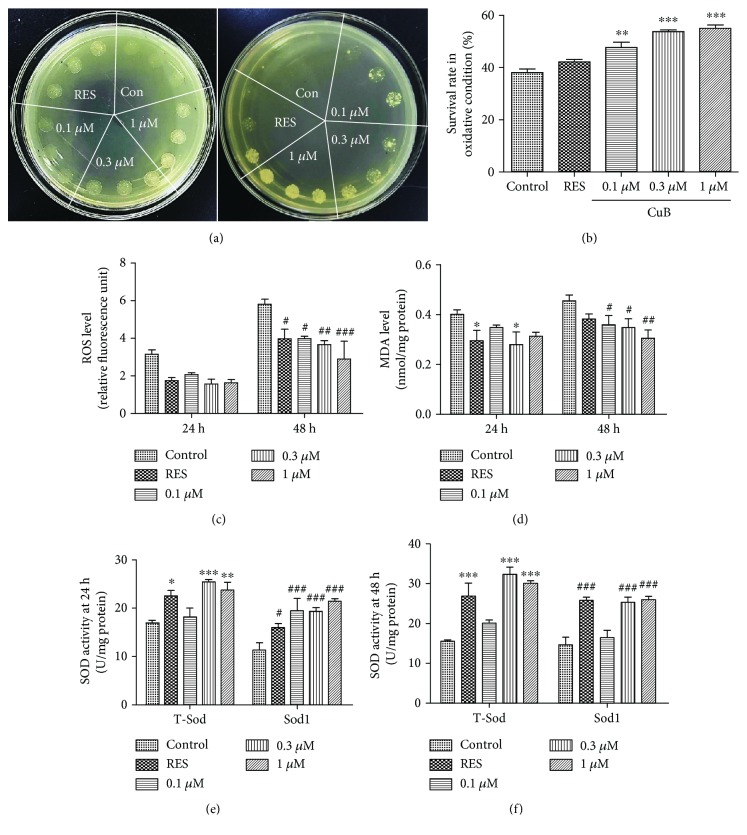
Effect of CuB on oxidative stress in yeast. (a) Effect of CuB on yeast growth with 5 *μ*L yeast culture and 1/10, 1/100, and 1/1000 dilutions under oxidative conditions simulated with 9 mM H_2_O_2_. (b) Effect of CuB on the survival rate of yeast under oxidative conditions simulated with 5 mM H_2_O_2_. ∗∗ and ∗∗∗ represent significant differences compared to the control group (*p* < 0.01 and *p* < 0.001, respectively). (c, d, e, f) Effect of CuB on ROS level, MDA accumulation, T-Sod, and Sod1 enzyme activity at 24 or 48 h. ∗, ∗∗, ∗∗∗ and #, ##, ### indicate significant differences from the corresponding control (*p* < 0.05, *p* < 0.01, and *p* < 0.001, respectively).

Oxidative stress is a major factor of aging and age-related diseases, and high levels of oxidative stress result in DNA damage, lipid peroxidation, and protein oxidation [28]. Therefore, we performed antioxidative experiments to determine whether antioxidative activity is involved in the antiaging effect of CuB. We used two methods to investigate the effect of CuB on the growth of yeast with 5 *μ*L culture or 1/10, 1/100, and 1/1000 dilutions of each group under oxidative stress at 9 mM H_2_O_2_. The growth of yeast in both conditions (Figure 3(a)) was significantly improved, respectively. We also examined the survival rate of yeast under oxidative stress and found that the survival rate was 38.09% ± 1.39 in the control group, 42.19% ± 0.87 in the RES group, 47.74% ± 2.03 in the 0.1 *μ*M CuB group (*p* < 0.01), 53.73% ± 0.73 in the 0.3 *μ*M CuB group (*p* < 0.001), and 55.01% ± 1.35 in the 1 *μ*M CuB group (*p* < 0.001) (Figure 3(b)). These results indicate that the regulation of antioxidative activity has an important role in the antiaging effect of CuB.

### 3.4. CuB Decreases ROS Accumulation and MDA Production and Increases Sod Activity

ROS are by-products of aerobic respiration and various metabolic processes. They cause secondary reactions, such as lipid peroxidation and protein oxidation [29]. MDA, a biomarker of lipid peroxidation in living cells, has cytotoxic effects [30]. Thus, we tested the change in ROS accumulation and MDA levels in CuB-treated yeast. ROS accumulation in yeast notably decreased after 48 h of incubation with 0.1, 0.3, and 1 *μ*M CuB (Figure 3(c)). Similarly, significant decreases were observed in MDA production by yeast. MDA production decreased after 24 h of incubation with 0.3 *μ*M CuB and 48 h of incubation with 0.1, 0.3, and 1 *μ*M CuB (Figure 3(d)). These results suggested that CuB could significantly inhibit the production of ROS and MDA in yeast.

Sod is a component of antioxidative defense systems that can scavenge ROS and other free radicals in cells. Yeast has two kinds of Sod, Sod1 and Sod2. Sod1 is a stable Sod that localizes in the cytoplasm, and mitochondrial Sod2 is more sensitive to environmental factors. Therefore, we evaluated total-Sod and Sod1 activity in yeast incubated with CuB for 24 or 48 h. As shown in Figures 3(e) and 3(f), T-Sod and Sod1 enzyme activity significantly improved after 24 and 48 h of treatment with 0.3 and 1 *μ*M CuB. Besides, Sod1 activity also improved after 24 h of treatment with 0.1 *μ*M CuB. The results show that CuB can significantly decrease ROS and MDA levels and increase T-Sod and Sod1 enzyme activity in yeast at 0.3 and 1 *μ*M CuB. These results suggest that CuB exerts its antiaging effect by regulating antioxidative activity.

### 3.5. CuB Does Not Affect the Replicative Lifespans of the Sod1, Sod2, Uth1, and Skn7 Mutants of K6001 Yeast

**Figure 4:**
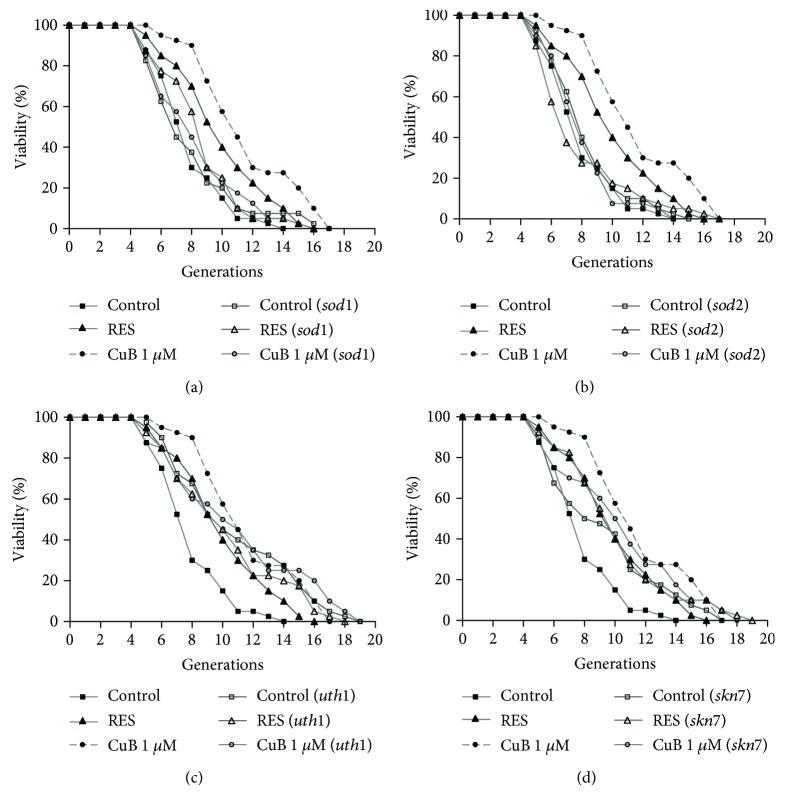
Effect of CuB on the replicative lifespans of *sod1* (a), *sod2* (b), *uth1* (c), and *skn7* (d) mutants with K6001 background. The procedure for the replicative lifespan assay was the same as that for the K6001 lifespan assay.

The results shown in Figure 3 suggest that antioxidative activity is involved in the antiaging activity of CuB and that *SOD1* and *SOD2* participate in oxidative stress responses. Therefore, we used *sod1* and *sod2* mutants with the K6001 background to investigate the involvement of *SOD1* and *SOD2* genes in the antiaging effect of CuB. As shown in Figures 4(a) and 4(b) and Table 2, the average replicative lifespan of the wild-type K6001 yeast was 6.95 ± 0.34 generations under the control treatment, 9.03 ± 0.47 generations under the RES treatment, and 9.60 ± 0.49 generations under the 1 *μ*M CuB treatment. The average replicative lifespan of the *sod1* mutant was 6.93 ± 0.43 generations under the control treatment, 7.73 ± 0.39 generations under the RES treatment, and 7.45 ± 0.46 generations under the 1 *μ*M CuB treatment. The average replicative lifespan of the *sod2* mutant was 7.35 ± 0.37 generations under the control treatment, 6.85 ± 0.47 generations under the RES treatment, and 7.10 ± 0.33 generations under the 1 *μ*M CuB treatment. These results show that the replicative lifespans of the *sod1* and *sod2* mutants are unaffected by RES or CuB treatment.

*UTH1* is an aging gene that is involved in the regulation of programmed cell death in yeast [31]. Skn7 is the transcriptional activator of *UTH1* and is associated with protection against oxidative stress [32]. In our previous study, *UTH1* gene expression in *skn7* mutant was significantly decreased [33]. To explore whether *UTH1* and *SKN7* genes are involved in the lifespan-extending effect of CuB, we measured the replicative lifespans of *uth1* and *skn7* mutants after CuB treatment. The average replicative lifespan of the *uth1* mutant was 9.95 ± 0.63 generations under the control treatment, 9.30 ± 0.59 generations under the RES treatment, and 9.90 ± 0.69 generations under the 1 *μ*M CuB treatment (Figure 4(c) and Table 2). The average replicative lifespan of the *skn7* mutant was 8.43 ± 0.58 generations under the control treatment, 9.13 ± 0.52 generations under the RES treatment, and 9.30 ± 0.60 generations under the 1 *μ*M CuB treatment (Figure 4(d) and Table 2). The replicative lifespans of the two mutants after CuB treatment did not significantly change. As the average replicative lifespan of the wild-type K6001 yeast was 6.95 ± 0.34 generations under the control treatment, the replicative lifespan of the *uth1* mutant was longer than that of the wild-type K6001. This result was consistent with other reports that deletion of *UTH1* increased yeast lifespan [34, 35]. These results indicate that *SOD1*, *SOD2*, *UTH1*, and *SKN7* are involved in the antiaging activity of CuB.

### 3.6. CuB Does Not Affect the Growth Nor Morphology of Yeast Cells

**Figure 5:**
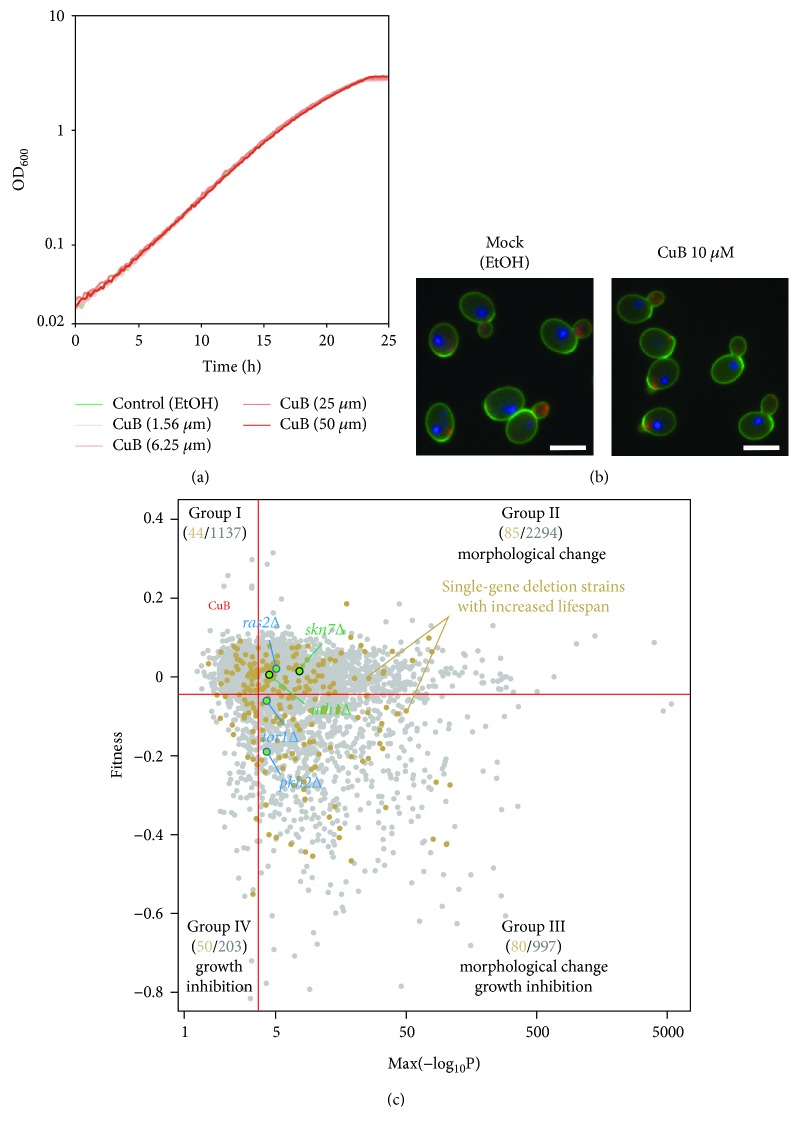
CuB neither inhibits cell growth nor causes morphological changes. (a) The growth curve of *Saccharomyces cerevisiae* in the presence and absence of CuB was plotted. The assay was repeated at least twice. (b) Images of cells treated with control (1% EtOH) or CuB (10 *μ*M). The fluorescent dyes fluorescein isothiocyanate-Con A, 4,6-diamidino-2-phenylindole, and rhodamine-phalloidin were used to stain the cell wall, nuclear DNA, and actin, respectively. Scale bar, 5 *μ*m. (c) Scatter plot of mutants with nonessential gene deletions in terms of specific morphological abnormality (*x*-axis) and growth rate (*y*-axis). The horizontal and vertical red lines indicate the false discovery rate of 0.01 for growth rate and morphology, respectively. The number shown in each area indicates the number of mutants categorized on the basis of cell growth and morphology (single-gene deletion strains with increased lifespan/mutants categorized on the basis of cell growth and/or morphology).

Many active compounds have been investigated in yeast with growth assay and morphological examination to understand their bioactivity. This is because both cell growth and morphology reflect numerous essential cellular processes, such as DNA replication, transcription, translation, vesicular transport, organelle assembly, and cell cycle regulation. Compared with the cells treated with solvent only (1% EtOH), treatment with 1.56 *μ*M to 50 *μ*M CuB failed to inhibit cell growth (Figure 5(a)). Since treatment with 0.1, 0.3, or 1 *μ*M CuB prolongs lifespan, cell growth is less affected by this drug. Examination of yeast morphology after fluorescent staining of the cell wall, actin, and nuclear DNA revealed that treatment with 10 *μ*M CuB did not cause obvious cell morphological changes (Figure 5(b)). After the fluorescent image pictures were quantified with a high-throughput, processing system CalMorph [19], we also statistically analyzed dose-dependent morphological changes with the Jonckheere-Terpstra test [36]. None of the 501 morphological parameters measured by CalMorph exhibited significant dose-dependent changes with FDR of 5%, confirming that CuB-treated cells did not change morphology significantly. These results suggested that CuB neither inhibits cell growth nor affects yeast morphology.

### 3.7. Potential CuB Targets

Characterization of nonessential gene deletion mutants in terms of cell growth and morphology can be used to classify genes into four groups: genes not responsible for growth and morphology (group I, 1137 genes), genes only important for morphology (group II, 2294 genes), genes required for both growth and morphology (group III, 997 genes), and genes only required for growth (group IV, 203 genes) (Figure 5(c)) [21]. We attempted to use this information for prediction of the drug target by assuming that inhibition of the gene product by the drug is equivalent to the functional defect by gene deletion. Since CuB neither affects growth nor morphology, CuB likely inhibits the gene function belonging to group I (Figure 5(c)). Our genetic evidence suggested that the aging-related genes, *UTH1* and *SKN7*, participate in antiaging effect of CuB. However, its interaction is likely indirect, because *UTH1* and *SKN7* belong to group II (Figure 5(c)). We also plotted gene deletion mutants with increased lifespan in Figure 5(c) [27]. Because group I contains 44 aging-related genes, we propose that CuB could target one of these 44 genes listed in Table 3. However, it cannot be excluded that the inhibition of the target by CuB is only partial, and that other genes, including essential ones, could be the main target of the drug.

## 4. Discussion

In China, CuB has been used to treat hepatitis for many years [37]. CuB also has potent anticancer, antioxidative, and anti-inflammatory activities [13, 14]. In the present study, we performed replicative lifespan assays to screen for antiaging substances from compounds library and used chronological lifespan assay to confirm the activity. We identified CuB as a potential antiaging compound. The chronological and replicative lifespan results shown in Figures 1(b) and 1(c) imply that CuB exerts significant antiaging effects in a dose-dependent manner.

We previously applied the replicative lifespan assay to evaluate the antiaging activities of other compounds. However, we found that this assay exhibits deficiencies that may affect the accuracy of its results. For example, random selection and subjective factors affect the quantification of daughter cells in the replicative lifespan assay. Therefore, to increase the accuracy of the bioassay results in the present study, we utilized the chronological lifespan assay to evaluate the antiaging activity of CuB. Yeasts have replicative and chronological lifespans. Replicative aging in yeast resembles the aging of mitotic cells in higher organisms, and chronological aging is similar to the aging of nondividing cells in higher eukaryotes [38]. The results of replicative and chronological lifespan assays suggest that CuB affects both the replicative and chronological lifespans of yeast.

In the present study, we focused on autophagy, oxidative stress, and longevity-related genes to investigate the mechanism of action of CuB. Autophagy levels decrease with age, and autophagy activation ameliorates age-related symptoms and has potential functions against aging [8]. Autophagy-related (*ATG*) or vacuolar protein-sorting genes encode some of the proteins that mediate autophagy [6]. Autophagy consists of nonselective and selective autophagy. Atg2 participates both nonselective and selective pathways of autophagy, including macroautophagy and pexophagy [39]; Atg32 is only essential for mitophagy [40]. Thus, we analyzed *ATG2* and *ATG32* expressions and performed replicative lifespan assays with *atg2* and *atg32* mutants with K6001 background and K6001 yeast and chronological lifespan assays with *atg2* and *atg32* mutants and YOM36 yeast to investigate the effect of autophagy on the antiaging activity of CuB. The results of gene expression, replicative lifespan, and chronological lifespan shown in Figures 2(a)–2(c) and 2(h) suggest that *ATG2* and *ATG32* genes have essential roles in the antiaging activity of CuB. Besides, enhanced mitophagy contributes to the extension of longevity by CuB.

Atg8 is an important component for autophagic machinery and participates in the whole process of autophagy, and it is a biomarker of autophagy in yeast [41]. Therefore, we investigated the effect of CuB on autophagy through detection of Atg8 with GFP-ATG8 fluorescent imaging and western blot analysis. Because RES can induce autophagy at 100 *μ*M in mammalian cultured cells [10], we used it as a positive control in experiments for detection of autophagy in the present study. The changes of GFP in yeast in Figures 2(d) and 2(e) and western blot results in Figures 2(f) and 2(g) suggest that CuB significantly induces the autophagy of yeast.

We also investigated the effect of antioxidative activity on the antiaging function of CuB. The changes in the survival rate and Sod, ROS, and MDA levels of yeast shown in Figure 3 demonstrate that CuB exerts its antiaging effect in yeast by regulating antioxidative activity.

Longevity-related and aging-related genes have important roles in the regulation of aging and longevity. *UTH1* is a yeast aging gene and takes part in oxidative stress [42]. *SKN7* is the transcriptional activator of *UTH1* and is related with protection against oxidative stress [32]. *SOD1* and *SOD2* genes encode Sod1 and Sod2 enzymes, respectively, which are important for redox homeostasis in cells. The replicative lifespan assay results of yeast mutants are shown in Figure 4 and Table 2 and reveal that *UTH1*, *SKN7*, *SOD1*, and *SOD2* genes are required in the lifespan-extension activity of CuB. In addition, the replicative lifespan of the *uth1* mutant is longer than that of the wild type. This result is consistent with the result of a previous study [35].

To predict the target of CuB, we used CalMorph to analyze the morphological changes exhibited by CuB-treated yeast cells. CalMorph is the image-processing software that has been used to analyze the morphology of 4718 nonessential gene mutants in more than 200 yeast strains. It can effectively identify compound targets [19]. The growth curve and morphological changes of cells treated with CuB are shown in Figure 5 and provide an important clue for the identity of the target genes of CuB. CuB does not cause growth inhibition nor cell morphological changes, and therefore, it could target one of the genes that are not involved in cell growth or morphology. After plotting mutations with long lifespan, we would pinpoint the possible targets of CuB among the 44 genes (Table 3). The results shown in Figure 5(c) suggest that CuB does not directly inhibit the genes mentioned in this study and that CuB might extend the lifespan of yeast by inhibiting upstream of aging-related genes mentioned in this study or by even activating some genes, such as *SIR2*, *SOD1*, and *SOD2*. If CuB activates some genes; activation of the CuB target would not cause growth inhibition or cell morphological changes. In this sense, the possible targets of CuB are not associated with cell growth or cell morphology. Nevertheless, additional efforts are needed to definitively identify the targets of CuB.

## 5. Conclusions

Overall, we found that CuB significantly prolongs the replicative and chronological lifespans of yeast in a dose-dependent manner. CuB exerts its antiaging effect by regulating autophagy and antioxidative activity. Moreover, the target identification of CuB suggests that 44 proteins that are not associated with cell growth or morphology are potential targets of CuB. However, the specific target of CuB requires further study, such as bioassay on these 44 proteins. Furthermore, given that CuB is already used to treat hepatitis cases in China, it may be developed as an antiaging drug.

## Figures and Tables

**Table 1:** Yeast strains used in the present study.

Strains	Genotype	Source
K6001	*MATa*, *ade2-1*, *trp1-1*, *can1-100*, *leu2-3*,*112*, *his3-11*,*15*, *GAL*, *psi*+, *ho::*HO*::CDC6* (at HO), *cdc6::hisG*, *ura3::URA3 GAL-ubiR-CDC6* (at *URA3*)	Gifted by Professor Michael Breitenbach
Δ*uth1* of K6001, Δ*skn7* of K6001, Δ*sod1* of K6001, Δ*sod2* of K6001, Δ*atg2* of K6001, Δ*atg32* of K6001	Replace the *UTH1* gene, *SKN7* gene, *SOD1* gene, *SOD2* gene, *ATG2* gene, and *ATG32* gene in K6001 with kanamycin gene, respectively	Constructed by Professor Akira Matsuura
BY4741	*MATa*, *his3*Δ*1*, *leu2*Δ*0*, *met15*Δ*0*, *ura3*Δ*0*	Gifted by Professor Akira Matsuura
YOM36	Prototrophic derivative of BY4742 (*MATα*, *his3Δ1*, *leu2Δ0*, *lys2Δ0*, *ura3Δ0*)	Gifted by Professor Akira Matsuura
Δ*atg2* of YOM36, Δ*atg32* of YOM36	Replace the *ATG2* gene and *ATG32* gene in YOM36 with kanamycin gene, respectively	Constructed by Professor Akira Matsuura
YOM38 containing plasmid pR316-GFP-ATG8	Prototrophic derivative of BY4742 (*MATα*, *his3Δ1*, *leu2Δ0*, *lys2Δ0*) containing plasmid pR316-GFP-ATG8	Constructed by Professor Akira Matsuura
YO2458	*MATa, his3Δ1, leu2Δ0, met15Δ0, ura3Δ0, YOR202w::KanMX4*	Provided by Professor Yoshikazu Ohya

**Table 2:** Replicative lifespan of K6001 and its mutants.

Figures	Yeast strains	Treatment (*μ*M)	Replicative lifespan (generations)
Figure 1(b)	K6001	Control	6.95 ± 0.34
		RES-10	9.03 ± 0.47^∗^
		CuB-0.1	8.90 ± 0.55^∗^
		CuB-0.3	8.95 ± 0.51^∗^
		CuB-1.0	9.60±0.49^∗∗^

Figures 2(b) and 2(c)	K6001	Control	7.05 ± 0.35
		RES-10	9.05 ± 0.55^∗^
		CuB-1.0	9.65±0.61^∗∗^
	*atg2* mutant of K6001	Control	6.95 ± 0.43
		RES-10	7.30 ± 0.40
		CuB-1.0	7.18 ± 0.45
	*atg32* mutant of K6001	Control	6.73 ± 0.42
		RES-10	7.40 ± 0.44
		CuB-1.0	7.10 ± 0.47

Figure 4	K6001	Control	6.95 ± 0.34
		RES-10	9.03 ± 0.47^∗^
		CuB-1.0	9.60±0.49^∗∗^
	*sod1* mutant of K6001	Control	6.93 ± 0.43
		RES-10	7.73 ± 0.39
		CuB-1.0	7.45 ± 0.46
	*sod2* mutant of K6001	Control	7.35 ± 0.37
		RES-10	6.85 ± 0.47
		CuB-1.0	7.10 ± 0.33
	*uth1* mutant of K6001	Control	9.95 ± 0.63
		RES-10	9.30 ± 0.59
		CuB-1.0	9.90 ± 0.69
	*skn7* mutant of K6001	Control	8.43 ± 0.58
		RES-10	9.13 ± 0.52
		CuB-1.0	9.30 ± 0.60

Replicative lifespan was shown as average ± SEM; ∗ and ∗∗ represent significant differences compared to the corresponding control groups (*p* < 0.05, *p* < 0.01.).

**Table 3:** The targets list of CuB predicted by CalMorph.

ORF	Gene	Description
YBL052C	SAS3	Something about silencing
YBR007C	DSF2	Deletion suppressor of mptfive/puffive mutation
YBR034C	HMT1	HnRNP methyl transferase
YBR042C	CST26	Chromosome stability
YDL093W	PMT5	Protein O-mannosyl transferase
YDL095W	PMT1	Protein O-mannosyl transferase
YDR006C	SOK1	Suppressor of kinase
YDR099W	BMH2	Brain modulosignalin homolog
YDR110W	FOB1	Fork blocking less
YDR313C	PIB1	Phosphatidylinositol(3)-phosphate binding
YDR486C	VPS60	Vacuolar protein sorting
YEL020C	PXP1	Peroxisomal protein
YER164W	CHD1	Chromatin organization modifier helicase and DNA-binding domains
YFR015C	GSY1	Glycogen synthase
YFR040W	SAP155	Sit4-associated protein
YGL006W	PMC1	Plasma membrane calcium
YGL079W	KXD1	KxDL homolog
YGL200C	EMP24	Endomembrane protein
YGR254W	ENO1	Enolase
YHL002W	HSE1	Has symptoms of class E mutants; resembles Hbp, STAM, and EAST
YIL002C	INP51	Inositol polyphosphate 5-phosphatase
YJL013C	MAD3	Mitotic arrest-deficient
YJL098W	SAP185	Sit4-associated protein
YKL098W	MTC2	Maintenance of telomere capping
YLR176C	RFX1	Regulatory factor X
YMR058W	FET3	Ferrous transport
YMR126C	DLT1	Defect at low temperature
YMR127C	SAS2	Something about silencing
YMR221C	FMP42	Found in mitochondrial proteome
YMR251W	GTO3	Glutathione transferase omega-like
YMR251W-A	HOR7	Hyperosmolarity-responsive
YNL142W	MEP2	Mourning's ends part II
YOL071W	SDH5	Succinate dehydrogenase
YOR311C	DGK1	Diacylglycerol kinase
YPR111W	DBF20	Dumbbell forming
YPL177C	CUP9	Homeodomain-containing transcriptional repressor
YBR054W	YRO2	Protein with a putative role in response to acid stress
YBR238C	YBR238C	Mitochondrial membrane protein
YIL089W	YIL089W	Protein of unknown function found in the ER and vacuole lumen
YCR101C	YCR101C	Putative protein of unknown function
YNL034W	YNL034W	Putative protein of unknown function
YDL172C	YDL172C	Dubious open reading frame
YDR048C	YDR048C	Dubious open reading frame
YGL165C	YGL165C	Dubious open reading frame

## Data Availability

All the figures and table used to support the findings of this study are included within the article.

## Supplementary Materials

Supplementary Materials Supplementary Fig. 1. The western blot analysis of GFP-ATG8 and free GFP of yeast after administrating CuB at different times and doses. Each experiment was independently repeated three times.
