# Supplementary material for: Cucurbitacin B Exerts Antiaging Effects in Yeast by Regulating Autophagy and Oxidative Stress
Source: Oxid Med Cell Longev. 2019 Jun 2;2019:4517091. doi: 10.1155/2019/4517091 (PMC6589324; doi:10.1155/2019/4517091)
Supplement: Supplementary Materials — Supplementary Fig. 1. The western blot analysis of GFP-ATG8 and free GFP of yeast after administrating CuB at different times and doses. Each experiment was independently repeated three times. [file 4517091.f1.docx]

**Supplementary information**

**Cucurbitacin B exerts antiaging effects in yeast by regulating autophagy and oxidative stress**

Yanfei Lin^1^, Yuki Kotakeyama^2^, Jing Li^1^, Yanjun Pan^1^, Akira Matsuura^3^, Yoshikazu Ohya^2^, Minoru Yoshida^4, 5^, Lan Xiang^1^*, Jianhua Qi^1^*

^1^College of Pharmaceutical Sciences, Zhejiang University, 866 Yu Hang Tang Road, Hangzhou, P. R. China

^2^Departments of Integrated Biosciences, Graduate School of Frontier Sciences, University of Tokyo, 5-1-5 Kashiwanoha, Kashiwa, Chiba 277-8562, Japan

^3^Department of Biology, Graduate School of Science, Chiba University, Chiba 263-8522, Japan

^4^Chemical Genomics research group, RIKEN Center for Sustainable Resource Science, 2-1 Hirosawa, Wako, Saitama 351-0198, Japan

^5^Department of Biotechnology and Collaborative Research Institute for Innovative Microbiology, The University of Tokyo, Yayoi 1-1-1, Bunkyo-ku, Tokyo 113-8657, Japan

Correspondence should be addressed to Jianhua Qi ([qijianhua@zju.edu.cn](mailto:qijianhua@zju.edu.cn)) and Lan Xiang ([lxiang@zju.edu.cn](mailto:lxiang@zju.edu.cn)).


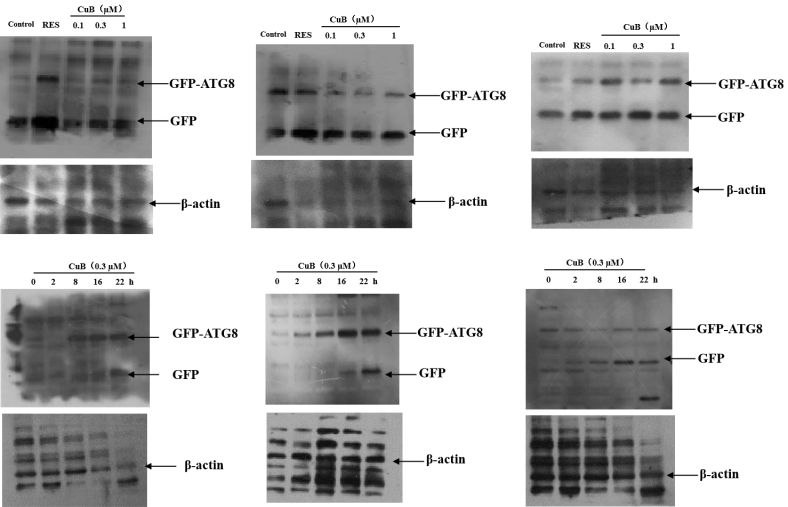


**Supplementary Fig. 1**. The western bot analysis of GFP-ATG8 and free GFP of yeast after administrating CuB at different time and doses. Each experiment was independently repeated three times.
